# Comparison of survival models and assessment of risk factors for survival of cardiovascular patients at Addis Ababa Cardiac Center, Ethiopia: a retrospective study

**DOI:** 10.4314/ahs.v21i3.29

**Published:** 2021-09

**Authors:** Belaynesh Yeniew Enyew, Zeytu Gashaw Asfaw

**Affiliations:** 1 Department of Statistics, DeberBirhan University, Debrebirhan, Ethiopia; 2 Department of Statistics, Hawassa University, Hawassa, Ethiopia

**Keywords:** Cardiovascular patient, survival analysis, non-parametric, semi-parametric, parametric

## Abstract

**Background:**

Cardiovascular diseases (CVDs) is disorders of heart and blood vessels. It is a major health problem across the world, and 82% of CVD deaths is contributed by countries with low and middle income. The aim of this study was to choose appropriate model for the survival of cardiovascular patients data and identify the factors that affect the survival of cardiovascular patients at Addis Ababa Cardiac Center.

**Method:**

A Retrospective study was conducted on patients under follow-up at Addis Ababa Cardiac Center between September 2010 to December 2018. The patients included have made either post operation or pre-operation. Out of 1042 cardiac patients, a sample of 332 were selected for the current study using simple random sampling technique. Non-parametric, semi-parametric and parametric survival models were used and comparisons were made to select the appropriate predicting model.

**Results:**

Among the sample of 332 cardiac patients, only 67(20.2%) experienced CVD and the remaining 265(79.8%) were censored. The median and the maximum survival time of cardiac patients was 1925 and 1403 days respectively. The estimated hazard ratio of male patients to female patients is 1.926214 (95%CI: 1.111917–3.336847; p = 0.019) implying that the risk of death of male patients is 1.926214 times higher than female cardiac patients keeping the other covariates constant in the model. Even if, all semi parametric and parametric survival models fitted to the current data well, various model comparison criteria showed that parametric/weibull AFT survival model is better than the other.

**Conclusions:**

The governmental and non-governmental stakeholders should pay attention to give training on the risk factors identified on the current study to optimize individual's knowledge and awareness so that death due to CVDs can be minimized.

## Background

Cardiovascular diseases (CVD) is an aggregation of disorders of the heart and blood vessels. Coronary heart disease, cerebrovascular disease, peripheral arterial disease, rheumatic heart disease, congenital heart disease, deep vein thrombosis and pulmonary embolism are collectively named as cardiovascular diseases (CVDs)^1^,^2^,^3^. It is the leading cause of mortality globally and more people died annually from CVDs than from any other cause 1,5,6,7,8. The burden of CVD is not evenly distributed, it varies throughout the world in type and distributions especially between developed and developing nations. An estimated 17.9 million people with CVD have died in 2016, which was 31% of all global deaths. Among the total deaths due to CVDs, over three quarters of CVD deaths were in low- and middle - income countries 9. Due to globalization, aging and accelerated urbanization, CVD is the leading cause of death in Ethiopia^10^. In order to reduce death of CVDs patients, adequate information on the distribution of risk factors in different geographic and socioeconomic groups of the population should be made. This is the ultimate goal and sole contribution of the current study. In fact, prevention of CVDs is always the most prioritized issues but equivalently, scholars should pay attention on the way to prolong the life of CVD affected patients. Appropriate intervention should be made so as to reduce mortality and morbidity due to CVD. All potential stakeholders are spending considerable time to identify the most important risk factors that could be a cause for death of cardiovascular patients. Authors of the current study reviewed the most common risk factors from literature 11,12,13,14. However, heterogeneity between patients is usually expected due to biological, environment, health facilities, physician experience and commitment difference. Thus, investigating new risk factors for the same disease in different geographic areas, individuals and time is necessary and it is the main concern of the current study. Besides to this, Addis Ababa cardiac center nationally it is the first in kind which were established by February 2009. But, its treatment effciency for prolonging the survival of patients not yet well investigated.

The survival of patient data usually make analysis through survival models. Survival analysis involve the modeling of time to event data and in the current study, death or failure is considered as an “event”^15^. In survival analysis literature traditionally only a single event occurs, after which the organism or mechanism is dead or broken. Several methods have been developed for the analysis of survival data such as Kaplan-Meier, Logrank test, Cox regression, Accelerated Failure Time (AFT), but due to complexity of data one may be popular than the others for predicting events 16,17,18,19. Thus, to make realistic analysis, there is a need to find-out the most appropriate statistical model and thus, model comparison also made.

Moreover, since considering the entire data set is challenging in terms of time, human resource and finance; the researchers are forced to consider samples to make inference about the population. The diffcult task here is obtaining the representative and optimal sample where non-statistician usually challenged to consider the appropriate sample size determination formula for survival data analysis. Therefore, in the current study the researchers introduce appropriate sample size determination procedures for survival data,

This paper is organized as follows. Section 2 describes the materials and methods.

The basic findings of the study are presented and discussed in Section 3. Finally, concluding remarks are provided in Section 4.

## Materials and Methods

The current study has considered secondary data from the cardiovascular patient's card and information sheet at Addis Ababa Cardiac Center.

### Study Population

The target population for the current study was cardiac patients who have taken either pre or post operations and who were under follow-up at Addis Ababa Cardiac Center from September 2010 − December 2018.

### Variables of the study

Several variables that supposed to associate with death of cardiovascular patients were considered for the current study.

### Dependent variable

The dependent variable of the current study is the survival time of cardiovascular patients. It is the time duration from the date of admission for treatment until date of death or censor. Cardiovascular patients who were alive during the study time or dropped before death were considered as censored. Right censoring was realized in the current data-set. Among the sample of 332 cardiac patients, only 67(20.2%) were observed and the remaining 265(79.8%) were censored. Usually, the survival models require the censoring percentage not exceeding to 50 percent 20. But in the current study the percentage of censoring was 79.8% which was higher than 50% because of the less observed data during the eight years follow-up. Such situation is frequently happening and several literature was used survival models while censored individual less than the expected one^21^.

### Independent variables (covariates)

Based on literature reviews, researchers experience and expertise suggestions, authors have considered the following explanatory variables: Age, Sex, Hypertension/Blood pressure, Dyslipidemia, Body mass index, Smoking, Alcohol use, Diabetic Milletus, Chest pain, Pulse rate, Educational status, Region, Income level, Leg swelling, Types of CVDs, Family history, Pericardium, Orthopnea and Patient status.

### Inclusion and Exclusion Criteria

The study has considered cardiovascular patients whose age was greater than or equals to 10 years and who have taken both pre and post operations, and who were under follow-up during the study period. However, the study excluded those patients whose age were less than 10 years and who have not taken either pre or post operations.

### Sampling Techniques and Sample Size Determination

In the current study, simple random sampling technique was used to select a representative sample from large number of cardiac patients. Unlike others statistical methods, in survival analysis the sample size determination procedures should considers the following facts: the null hypothesis to be tested, test statistic, assumed effect size, size of the test (significance level, α), desired power, sample size (in terms of number of events), probability of an event during study, expected rate of loss, Sample size (in terms of number of cardiac patients) and adjustments for interim analysis. The present study considered hazard ratio from previous study on cardiovascular patients in Ethiopia. For this study, we have used (power = 1 − β), level of significance - α, type II error (β) and Equal allocation (π1= π2=½) for variable sex (male and female groups) of cardiac patients. Thus, total number of cardiac patients can be calculated as:

$$\text{n}=\frac{\text{events}}{\mathrm{Pr}(\text{events})},\text{ events}=\frac{{\left(z_{\alpha /2}+z_{\beta }\right)}^{2}}{{\text{π}}_{1}{\text{π}}_{2}{\left(\log\text{HR}\right)}^{2}}$$

Where,

zα/2 and zβ are standard normal percentiles 22 and the values are 1.96 at α = 0.05 level of significant and 0.842 at β =0.2 with 0.8 desire power respectively, HR= 0.32 (the hazard ratio of male patients to female cardiac patients) and pr(events) = 1 − π1S1(T) − π2S2(T). events=(1.96+0.842)2 0.5 *0.5(log(0.32))2 = 128.688525

We have values for S1 (T) and S2 (T) by assuming Exponential Survival Times. For exponential failure times, IR= λ and S(t) = exp(−λt). The researcher uses an assumed IR to calculate failure probability for Sample Size calculations. Eight years (T= 8), equal allocation estimate of IR for one group is 0.8 events / p-eight year (or 0.1 events / person-one-year). For power calculations:

Pr (event) = 1 – (0.449328996+ 0.77414197)/2 = 0.3882645


$$\text{n}=\frac{\text{events}}{\mathrm{Pr}(\text{events})}=\frac{\text{128.688552}}{\text{P0.3882645}}=\text{331.445579 = 332}$$

Hence, after we got the optimal sample size, we have used simple random sampling technique to select the desired sample from a total of 1042 cardiac patients.

### Survival Models

Survival data or time to event data measure the time elapsed from a given origin to the occurrence of an event of interest. In survival analysis, the researcher usually refers the time variable as survival time because it gives the time that an individual has ‘survived’ over some follow-up period 23. There are three primary approaches to model survival processes: Non-parametric, Semi-parametric and Parametric survival models.

### Non-parametric Survival Models

Non-parametric analysis are widely used in situations where there is doubt about the exact form of distribution. Survival data are conveniently summarized through estimates of the survival function and hazard function. However, the distribution might not be perfectly pinned down mathematically. The estimation of the survival distribution provides estimates of descriptive statistics such as the median survival time. Median survival time is better than mean survival due to the fact that, it is not dependent on all the times to event being known. On the other hand, the mean time to event requires that all times to events are known but this is not the case all the time due to censoring problem. Moreover, the distribution of survival time is skewed and thus, it is described usually using median.

In the current study, Kaplan-Meier estimator was used to estimate the survival probability of cardiovascular patients and log rank test was used for comparison of survival of patients in different categories^24^. If the Kaplan-Meier estimator for the whole observations period is more than 50%, the median survival time cannot be determined.

### Semi-parametric Survival Models

The Cox regression model^25^ is semi parametric survival model where the baseline hazard takes no particular distribution. It is still the more preferable than parametric survival models because it has broad versatility and it contains both parametric and non-parametric parts simultaneously. h(*t*) = λ_0_(*t*)*exp*(*β*0X), where, λ_0_(*t*) is baseline hazard (the hazard value when the value of all covariates is zero).

### Parametric Survival Models

Parametric survival models usually assume some shape for the hazard rate (i.e. flat, monotonic, etc). Usually hazard rate are expressed as a function of covariates *h_i_*(*t*) = *g*(*x_i_β*), and interpreted as the change in X. When all the covariates equals to zero, *h_i_*(*t*) = *g*(*β*_0_), the base line hazard. Among the popular parametric survival regression models, authors have considered Weibull, Exponential, Log-normal and Log-logistic. An advantage of using a parametric distribution, it is possible to predict time to event well after the period during which events have occurred for the observed data.

### Weibull and Exponential models

The Weibull and exponential models are parametrized as both Proportional hazard (PH) and Accelerated Failute Time(AFT) models. The Weibull distribution is suitable for modeling data with monotone hazard rates that either increase or decrease exponentially with time, whereas the exponential distribution is suitable for modeling data with constant hazard. For the PH model, *h*(*t*) = *λ*, for exponential regression, and *h*(*t*) = *λpt*^*p*−1^ for Weibull regression, where λ is the shape parameter to be estimated from the data. Some authors refer not to λ but to $\sigma =\frac{1}{p}$ 26.

### Log-normal and Log-logistic models

The log-normal and log-logistic models are implemented only in the AFT form. These two distributions are similar and tend to produce comparable results. For the log-normal distribution, the natural logarithm of time follows a normal distribution; for the log logistic distribution, the natural logarithm of time follows a logistic distribution. The log-normal survivor function is given by:

$$S(t)=1-\varphi \left(\frac{\log(t)-\mu }{\sigma }\right)$$

where φ(z) is the standard normal cumulative distribution function. The log-normal regression is implemented by setting *μ_j_ = x_j_β* and treating the standard deviation, σ, as an ancillary parameter to be estimated from the data. The log-logistic regression is obtained if zj has a logistic density. The log-logistic survivor function is given by:

$$S(t)={\left(1+{\left(\lambda t\right)}^{\frac{1}{\gamma }}\right)}^{-1}$$

This model is implemented by parameterizing *λ_j_ = exp* (−*x_j_λ*) and treating the scale parameter γ as an ancillary parameter which is estimated from the data^22^.

## Results

Summary of Demographic, Socio-economic and Environmental covariates of 332 CVD patients who were under follow-up in Addis Ababa Cardiac Center are presented in Table 1.

**Table 1:** Summary of Demographic, socio-economic and Environmental Excovariates of CVD Patients

Covariates	Categorie	Event (%)	Censored (%)	Total (%)
	Female	27(16.3%)	139(83.7%)	166(50%)
Sex	Male	40(24.1%)	126(75.9%)	166(50%)

Age at start	10–20	29(18.1%)	131(81.9%)	160(48.2%)
treatment	20–30	18(25.4%)	53(74.6%)	71(21.4%)
	≥30	20(19.8%)	81(80.2)	101(30.4%)

	No	31(26.1%)	88(73.9%)	119(35.8%)
Education	Yes	36(16.9%)	177(83.1%)	213(64.2%)

	AddisAbeba	31(22.3%)	108(77.7%)	139(41.9%)
Region	Oromia	14(15.4%)	77(84.6%)	91(27.4%)
	Other regions	22(10.9%)	180(89.1%)	102(30.7%)

Economical Level	Below Average	46(22.8%)	162(80.2%)	208(62.65%)
	Average	16(17.4%)	76(82.6%)	92(27.71%)
	Above Average	5(15.6%)	27(84.4%)	32(9.63%)

Smoking use	No	44(16.9%)	216(83.1%)	260(78.3%)
	Yes	23(31.9%)	49(68.1%)	72(21.7%)

Alcohol use	No	29(13.8%)	181(86.1%)	210(63.3%)
	Yes	38(31.1%)	84(68.9%)	122(36.7%)

Types of CVDs	CRHD	25(37.3%)	42(62.7%)	67(20.2%)
	CHD	20(28.6%)	50(71.4%)	76(22.9%)
	PAD	5(12.2%)	36(87.8%)	41(12.3%)
	ASD	5(11.4%)	39(88.6%)	44(13.3%)
	CDA	3(16.7%)	15(83.3%)	18(5.4%)
	Others	9(10.5%)	77(89.5%)	86(25.9%)

	Low	45(25.4%)	132(74.6%)	177(53.3%)
Blood Pressure	Normal	5(5.2%)	91(94.8%)	96(28.9%)
	High	17(28.8%)	42(71.2%)	59(17.8%)

Dyslipidemia No	38(16.7%)	190(83.3%)	228(68.7%)
	Yes	29(27.9%)	75(72.1%)	104(31.3%)

Pulse Rate	Regular	28(12.1%)	203(87.9%)	231(69.6%)
	Irregular	39(38.6%)	62(61.4%)	101(30.4%)

	Under weight	21(38.9%)	33(61.1%)	54(16.3%)
Body Mass Index	Normal	24(13.6%)	152(86.4%)	176(53%)
	Over weight	22(21.6%)	80(78.4%)	102(30.7%)

Family History	No	45(18.8%)	194(81.2%)	239(72%)
	Yes	22(23.4%)	71(76.3%)	93(28%)

Orthopnea	No	38(17.0%)	185(83.0%)	223(67.2%)
	Yes	29(26.6%)	80(73.4%)	109(32.8%)

Leg swelling	No	52(20.2%)	198(79.2%)	250(75.3%)
	Yes	15(18.3%)	67(81.7%)	82(24.7%)

Chest pain	No	25(12.4%)	177(87.6%)	202(60.8%)
	Yes	42(32.3%)	878(67.7%)	130(39.2%)

Diabetic Mellitus	No	33(14%)	202(86.0%)	235(70.8%)
	Yes	34(35.1%)	63(64.9%)	97(29.2%)

	Not active	36(31.0%)	80(69.0%)	116(34.9%)
Pericardium	Active	31(14.4%)	185(85.6%)	216(65.1%)

Dyspnea	No	40(20.0%)	161(80.0%)	201(60.5%)
	Yes	27(20.6%)	104(79.4%)	131(39.5%)

Median survival time of CVDs patients in the current study was 1925 days. This median value indicates that half of the patients died with probability 0.5. Among 332 CVD patients, 166 (50%) were females and 166 (50%) were males. The proportion of death for male patients was 24.1% which was greater than that of female patients 16.3%. Since Addis Ababa cardic center is located in Addis Ababa, majority of patients were from Addis Ababa and others from the surrounding area, oromia region. Hence, regional distribution elaborates that 41.9% of patients were from Addis Ababa, 27.4% of patients were from oromiya region and 30.7% were from other regions. The death proportion for Addis Ababa was 22.3% which was larger than both Oromia (15.4%) and other (10.9%). Considering educational status of cardiac patients, 35.8% of them were uneducated and 64.2% of them were educated. The death proportion was higher for uneducated patients (26.1%) compared to educated patients (16.9 %). This indicates that education can matter to be affected by CVD. Obviously, majority of Ethiopian not yet well educated and this mean that, the prevalence of CVD in Ethiopia will be high. Thus, appropriate training should be given for uneducated people to optimize awareness about CVD and clearly identified the risk factors that exposed them to CVD.

Pertaining to cardiovascular diseases distribution, 20.2% of them were Chronic rheumatic heart disease(CRHD), 22.9% of them were Congenial heart disease(CHD), 12.3% of them were Patient ducts arterious(PAD), 13.3% of them were Atial septal defect(ASD), 5.4% of them were Coronary artery disease(CDA) and the rest 25.9% of them were other types of CVDs. The death proportion was higher for those patients who had CRHD (37.3%), followed by those who had CHD (28.6%), CDA (16.7%), PDA (12.2%), ASD (11.4%) respectively, while the lowest proportion of death (10.5%) was among other types of CVDs. Regarding to blood pressure (BP), 177(53.3%) of the patients had low BP, 96(28.9%) of them were normal and 59(17.8%) of the had high BP. The death proportion was higher for those patients who had high BP (28.8%), followed by those patients who had low BP (25.4%), while people who have normal BP show the lowest proposition of deaths (5.2%).

Out of the total cardiac patients, only 93(28%) of the patients had family history of cardiac disease but 239(72%) of them had no such history. The death proportion was higher for patients who had family history of cardiac disease (23.4%) than those who had no history (18.8%). Vis-à-vis diabetes mellitus, 235(70.8%) of the patients were not affected with diabetes mellitus and 97 (29.2%) of them were affected with diabetes mellitus. From this, the death proportion was higher for patients with diabetes mellitus (35.1%) and lower (14%) for patients without diabetes mellitus. This means that family history and positive with diabetes mellitus could aggravate deaths of CVD patients.

Regarding pericardium, 116(34.9%) of the cardiac patients had no active pericardium and 216(60.1%) of them had active pericardium. The death proportion was higher for those patients who had no active pericardium(31%) and lower for patients who had active pericardium (14.4%). Likewise, 201(60.5%) the patients had dyspnea (shortness of breath) problem and 131(39.5%) of them had no dyspnea problem. The death proportion seems lower for patients without dyspnea (20.0%) and higher for patients with dyspnea (20.6%).

Comparison of Survival Estimates of Different Categories of Covariates Using Kaplan-Meier Survival Curve The Kaplan-Meier survival curve used to compare the survival of cardiac patients under different categories of categorical covariates. In general, patients belongs to the categories whose survival curve lays above the survival curve of the other category has a better survival time.

In this regard, the Kaplan-Meier survival curve revealed that patients who drink alcohol and smoking cigarettes had less survival time than those patients who do not drink alcohol and smoke cigarettes respectively. Patients who were affected by CRHD had less survival time as compared to those who were affected by CHD, CAD, PDA, ASD and Others types of CVDs. Similarly, patients who were affected by CHD had less survival time as compared to patients who were affected by CAD, PDA, ASD and Others types of CVDs, and patients who were affected by CDA had less survival time as compared to patients that were affected by PDA, ASD and other types CVDs. In the same manner, patients who were affected by PDA had less survival time as compared to patients that were affected by ASD and Others types CVDs, and patients who were affected by ASD had less survival time as compared to patients that were affected by Others types CVDs. In the same way, survival of cardiac patients who were overweight had less survival time than cardiac patients who were underweight and normal, and cardiac patients who were underweight had less survival time compared to patients who were normal. Likewise, the survival time of cardiac patients without orthopnea was less than those cardiac patients with orthopnea.

Comparison of Survival Estimates among catagories in the catagorical covariates Using Log-rank test The difference in survival time of categorical covariates is also supported by the log-rank test. As it is indicated in Table 2, sex, educational status, smoking, alcohol use, types of CVDs, blood pressure, pulse rate, body mass index, dyslipidemia, orthopnea, diabetes mellitus, chest pain and pericardium were significant covariate, whereas age, region, economic level, leg swelling and family history of patients were not significant at 5% level of significant.

**Table 2:** Comparison of Survival Curves among covariate through Log-rank test

Covariates	Chi-square value	Df	p-value
Sex	5	1	0.02527
Age	1.4	2	0.496
Educational status	8.9	1	0.00282
Region	1.2	2	0.854
Economic level	0.9	2	0.638
Smoke use	6.5	1	0.0105
Alcohol use	13.5	1	0.000238
Types of CVDs	20.2	5	0.00115
Blood pressure	16.1	2	0.00032
Dyslipidemia	4.2	1	0.0396
Pulse rate	22.3	1	2.27e-06
Body Mass Index	12.7	2	0.00175
Chest pain	11.4	1	0.000738
Orthopnea	4.9	1	0.0269
Leg swelling	0.1	1	0.871
Family History	1.5	1	0.215
Diabetic Millitus	5.3	1	0.0217
Pericardium	6.5	1	0.0108
Dyspnea	0.3	1	0.573

Authors of the current study used Multivariable survival analysis instead of univariate analysis to consider the possibility that a weakly associated variables could become an important predictor of the outcome when taken together 27. Thus, model comparisons have made and significant risk factors are selected based on the most preferred model.

The results of the multivariable cox proportional hazard model in Table 3 showed that sex, educational status, types of CVDs, blood pressure, pericardium, alcohol use, pulse rate, chest pain and family history of cardiac disease were significant covariates at 0.05 level of significance. Hence, these covariates had significant effect on the survival of cardiovascular patients as it was also shown in the Log-rank test. The the researchers include only the main effects in multivariable model none of the interactions between covariates were significant. Parametric Survival Models QQ plot, AIC and Log-likelihood were used to identify the appropriate parametric survival models among the four widely considered parametric survival models. Thus, the researcher used Weibul survival model to determine predictors of CVD patient since it has smaller AIC and Loglikelihood as shown in Table 4.

**Table 3:** Multivariable Cox PH regression Analysis

Covariates	categories	coef	se(coef)	Chi-squ	exp(coef)	P-value	95% of CI	
							Lower	Upper
Sex	Female (re)				1			
	Male	0.77394	0.29063	2.663	2.1683	0.007746 **	1.22668	3.8327

Education	Uneduc(re)				1			
	Educated	-0.91016	0.30041	-3.030	0.4025	0.002448 **	0.22336	0.7252

Pulse rate Regular(re)				1			
	Irregular	0.97069	0.29467	3.294	2.6398	0.000987 ***	1.48163	4.7032

Blood	Lower BP (re)				1			
Pressure	Normal BP	-1.93459	0.54545	-3.547	0.1445	0.000390***	0.04961	0.4208
	High BP	0.12515	0.35782	0.350	1.1333	0.726512	0.56206	2.2852

Family history	No FH (re)				1			
	FH	0.65236	0.30999	2.104	1.9201	0.035340 *	1.04580	3.5252

Alcohol use	No (re)				1			
	Yes	0.89163	0.29326	3.040	2.4391	0.002363 **	1.37280	4.3336

Pericardium	No(re)				1			
	Yes	-0.88867	0.29871	-2.975	0.4112	0.002929 **	0.22898	0.7384

Types of CVDs	CRHD(re)				1			
	CHD	-0.71041	0.34524	-2.058	0.4914	0.021038 *	0.24981	0.9668
	PDA	-0.87376	0.54755	-1.596	0.4174	0.110541	0.14271	1.2207
	ASD	-1.04366	0.52130	-2.002	0.3522	0.045283 *	0.12677	0.9783
	CAD	-1.53866	0.73080	-2.105	0.2147	0.035253 *	0.05125	0.8991
	Others	-1.33200	0.45119	-2.952	0.2639	0.003155 **	0.10901	0.6391

Chest pain	No(re)				1			
	Yes	0.47784	0.29632	1.613	1.6126	0.106837	0.90218	2.8824

**Table 4:** Parametric Survival Models

Model Type	Df	AIC value	Log-likelihood
Exponential	23	1155.747	-553.9
Weibull	22	1142.499	-547.2
Log-logistic	22	1143.318	-547.7
Log-normal	22	1146.725	-549.4

**Figure 2:**
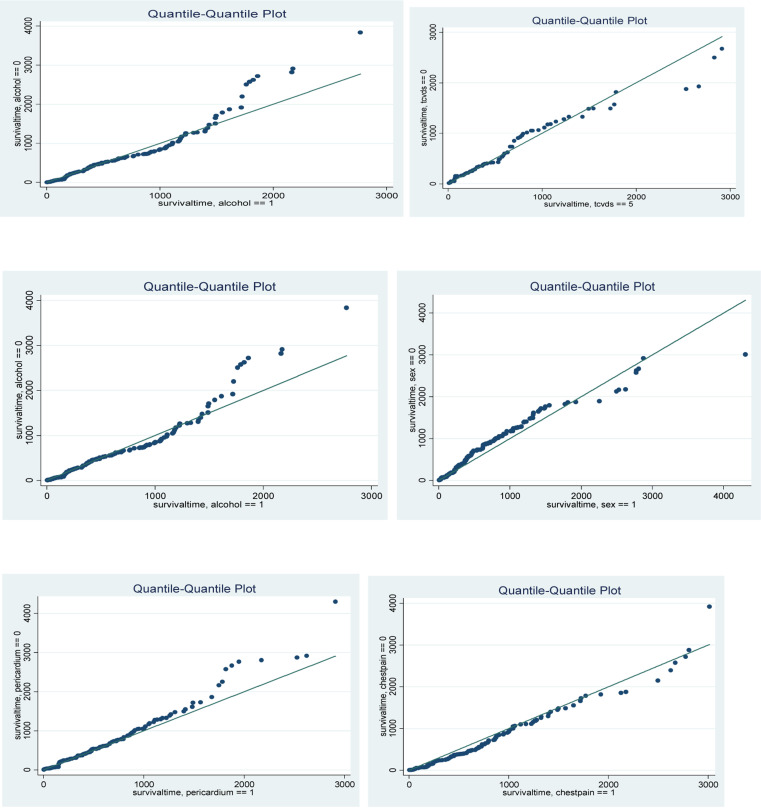
Quantile-Quantile Plot.

Quantile-Quantile Plot A quantile-quantile plot was made to check whether the accelerated failure time model provide an adequate fit to the data set or not. Authors checked the adequacy of the accelerated failure-time model by comparing the various catagories, Figure 2. The figures are approximately linear for all covariates. Hence, the Weibul accelerated failure time model has better performance.

The researchers have also checked the goodness of fit of the weibull model based on the likelihood ratio test. As shown in Table 5, the full model is better than the null model at 0.05 level of significance.

**Table 5:** Results of the Model Fit

Model	Model fitting Criterion		Likelihood ratio test		
	
	-2 Log Likelihood	AIC	Chi-Square	Df	Sig.
Null model	1222.6	1226.662	-	-	-
Full model	1118.4	1142.499	104.35	16	5.3e-15

The results of Weibull AFT model presented in Table 6 showed that explanatory variables, sex, family history, educational status, types of CVDs, BP, PR, alcohol use and pericardium have significant effect on survival of CVDs patients at 5% levels of significance similar to the cox model.

**Table 6:** Weibull AFT Model

Covariates	Categories	HR	se(*β̂*)	Z	P-value	95% CI	

						Lower	upper
Sex	Female (re)			1			
	Male	1.926214	.5400084	2.34	0.019	1.111917	3.336847

Family history	No FH (re)						
	FH1	2.074481	.6211866	2.44	0.015	1.153517	3.730739

Pulse rate	Regular PR(re)			1			
	Irregular PR	2.538132	.6908861	3.42	0.001	1.488727	4.327265

Types of CVDs	CRHD(re)			1			
	CHD	0.5960459	0.1939821	-1.59	0.112	1.127981	1.127981
	PDA	0.4627745	0.2506541	-1.42	0.155	.1600775	1.337853
	ASD	0.357208	0.1871871	-1.96	0.049	0.1278991	0.9976423
	CAD	0.258471	0.1696416	-2.06	0.039	.0714077	0.935575
	Other CVDs	0.2548515	0.1060211	-3.29	0.001	0.1127659	0.5759659

Educational status	Uneducated(re)			1			
	Educated	0.3952112	0.1095771	-3.35	0.001	.2295215	.680511

Chest pain	No(re)			1			
	Yes	1.839636	0.4998592	2.24	0.025	1.080057	3.133411

Alcohol use	No(re)			1			
	Yes	2.220152	0.5851375	3.03	0.002	1.32447	3.721544

Pericardium	Not Active(re)			1			
	Active	0.4044855	0.1145168	-3.20	0.001	0.2322272	0.704519

Blood pressure	Low BP(re)			1			
	Normal BP	0.181415	0.0910562	-3.40	0.001	0.0678323	0.485188
	High BP	1.084661	.3473742	0.25	0.800	0.579012	2.03189

Model Comparison and Selection One wants to select the better model among several choices based the performance of the models for the following reasons. First, people can understand simpler models with fewer predictors and less complicated structure. Second, one can certainly add more and more features into the model without screening and get better and better fit, till perfect fit is achieved, but the problem is over fitting. The interest of the authors is to find the best-predicting model not the best fitting model.

Table 7 shows the standardized variability of the cox and the weibull models on significant covariates. The model with least value of standardized variability and higher hazard ratio fits the data well. The value of standardized variability of all covariates were minimum, hazard values higher and AIC value is smaller in weibull model relative to cox proportional model. Thus, researchers conclude that the Weibull regression model is the most appropriate model for predicting the current cardiac data set.

**Table 7:** Cox and Parametric Models of Survival Cardiac Patients in Multivariate Analysis

Covariates	Cox model		Weibull AFT model	
	Standardized Variability	AIC	Standardized Variability	AIC
Sex	0.453(HR=1.7392)	618.42	0.479(HR=1.6829)	374.18
Educational status	0.342 (HR=0.4829)	615.15	0.326(HR=0.4744)	369.88
Pulse rate	0.225 (HR=3.0526)	603.023	0.216(HR=3.1494)	357.01
Blood pressure	21.8 (HR= 0.9924)	623.41	4.994(RR=0 .9683)	378.61
Family history	0.83(HR=1.3798)	621.95	0.7821 (HR=1.3950)	377.08
Types of CVDs	0.262(HR=.07495)	606.85	0.267(HR=0.754)	362.70
Alcohol use	0.281(HR=2.4647)	610.54	0.2775 (HR=2.4405)	365.55
Chest pain	0.305(HR=2.2919)	612.19	0.2999(HR=2.3232)	367.02
Pericardium	0.3986 (HR=0.5372)	617.135	0.4068(HR=0.5459)	372.60

## Discussion

Non-parametric, semi-parametric and parametric survival analysis were used to examine the factors affecting the survival of cardiovascular patients. The analysis revealed that some demographic, socioeconomic and environmental factors had statistically significant effect on the survival of cardiovascular patients. Based on weibull model the covariates influencing survival of cardiovascular patients were Sex, Types of CVDs, Education status, Blood Pressure, pulse rate, Alcoholic use, pericardium, chest pain and Family history of cardiac disease. The findings in the current study are also comparable with some other studies earlier.

Half of all CVDs patients under follow-up were male and the other half were female. However, Female cardiac patients had low proportion of deaths than the male patients. This result contradicts with earlier finding 28, who reported that the percentage of deaths is higher for women. But, it is consistent with the study conducted at Washington which stated that male patients had lower survival time (higher hazard rate) as compared to women patients 29, 30

According to the present study, the risk of death of alcohol user cardiovascular patients is higher as compared to non-user cardiac patients. This study also revealed that, blood pressure had significant effect on the survival of cardiovascular patients. Patients with normal blood pressure had less risk of death (high survival) as compared to those cardiac patients having abnormal blood pressure (high or low blood pressure). A similar finding^29^,^32^, suggested that blood pressure is a well-known risk factor for cardiovascular diseases. And another similar study on cardiac patients at Tikur Anbesa Specialized Tertiary Referral Hospital using cox regression model identify blood pressure as one of the major significant factors that affect the survival of cardiac patients 31.

In the current study, age of patients at start of treatment and body mass index had no significant effect on the death of cardiovascular patients, but pulse rate and types of CVDs had significant effect on the survival of cardiovascular patients. This finding contradicted with the finding of earlier study^14^ which suggested that age and body mass index had significant effect on the death of cardiovascular patients, but pulse rate and types of CVDs have no significant effect on the survival of cardiovascular patients. This result also contradict with the previous results 33, which stated that cardiovascular system is strongly affected by ageing.

Based on the current study, the variable pericardium had a significant effect on the survival of cardiovascular patients, cardiac patients having active pericardium has more survival compared with cardiac patients having no active pericardium.

The present study stated that, family history of cardiac diseases had significant effect on the survival of CVDs patients. Cardiovascular patients who have family history experienced less survives time than those patients who have no any family history. This finding is consistent with the report of earlier study 34 which states that family history of cardiac diseases was the main cause of cardiovascular disease and cardiac patients inherit heart diseases with higher tendency.

Based on this finding, chest pain has a significant effect on the survival of cardiac patients. Cardiovascular patients with chest pain had less survival time(higher hazard) than those cardiac patients without chest pain. Moreover, this study revealed that cardiovascular diabetes mellitus had no statistically significant effect on the survival of cardiac patients. Contrary to the current study 35 states that Diabetes mellitus is an important chronic disease on CVD morbidity and mortality. In addition, 36, 37 contradict to the current finding.

## Conclusions

The median survival time of cardiac patients at Addis Ababa Cardiac Hospital is 50% and this means that it needs to be optimized to 80% so that majority of patients will survive longer. Survival of CVDs patients was determined by their sex, types of CVDs, pulse rate, blood pressure, chest pain, family history of cardiac disease, educational status, pericardium and alcohol use. Although both semi parametric and parametric survival model has given similar significant factors, the parametric model(weibull AFT model) predict well to the cardiac data set even from other parametric models. Health extension programs should be implemented on a nationwide basis in Ethiopia, in order to inform policy and develop strategies and control risk factors of survival of cardiovascular patients. Thus, governmental and non-governmental organization should pay attention to give training on the risk factors identified on the current paper so as to create awareness and to reduce death CVDs patients.

## Figures and Tables

**Figure 1:**
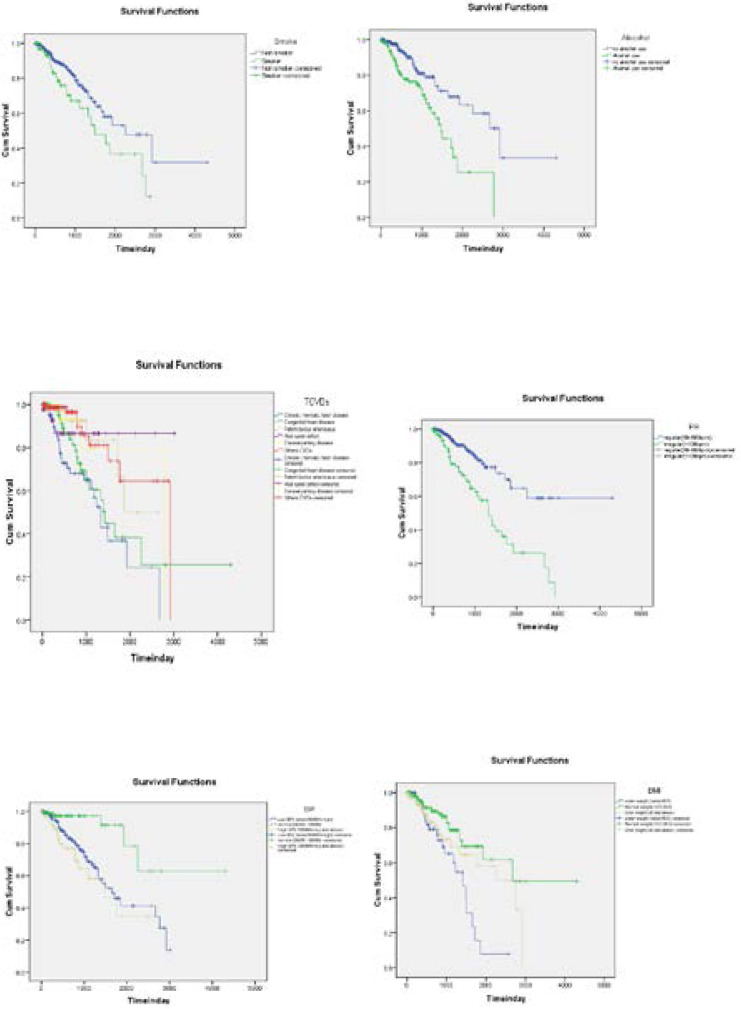
Kaplan-Meier Survival Curves among Covariates.

## Abbreviations

AIC:Akaike Information Criteria CVD:Cardiovascular disease BMI:Body Mass Index HR:Hazard Ratio AFT:Accelerated frailer time PH:Proportional Hazard BP:Blood Pleasure CRHD:Chronic rheumatic heart disease CHD:Congenial heart disease

## Avaliability of data and materials

Raw data can be made avaliable on request to the corresponding author.

## Limitations of the study

We were unable to include important socio-demographic and socioeconomic factors like, physical activity, diet types, cholesterol level, and place of residence, ethnicity and others that might have contribution on the survival times of CVD patients. Globally, these factors are the major causes of death that have increased substantially since 1990 in some large populations and thus, such variables never been missed in any death related studies.
